# Cellulose Beads Derived from Waste Textiles for Drug Delivery

**DOI:** 10.3390/polym12071621

**Published:** 2020-07-21

**Authors:** Beini Zeng, Xungai Wang, Nolene Byrne

**Affiliations:** Institute for Frontier Materials, Geelong Waurn Ponds Campus, Deakin University, Waurn Ponds 3216, Victoria, Australia; xungai.wang@deakin.edu.au

**Keywords:** porous cellulose, porous structure, textile recycling, waste utilisation, drug delivery

## Abstract

Cellulose beads were successfully prepared from waste denim using a dissolution-regeneration approach with ionic liquids as the dissolving solvent. Cellulose beads with different morphologies were achieved by altering the dissolving and coagulating solvents. The morphological differences were quantified by N_2_ physisorption. The impact of morphology on the cellulose beads’ potential application was investigated in the context of drug loading and release. The results show that the fibrous morphology showed a better loading capacity than the globular analogue due to its higher surface area and pore volume.

## 1. Introduction

The negative environmental impacts of the textile industry have been in the spotlight in recent years due to the increasing public awareness around sustainability and climate change. One of the pressing issues faced by the textile industry is the increasing amount of textile waste [1,2]. Global textile fibre production has reached 150 million tonnes, and 92 million tonnes of solid waste is generated annually [3]. Cotton is extensively utilised in the textile industry and accounts for 24% of the total textile fibre production [4]. It is also the dominating component of the largest fashion item on the market, denim. Hence, denim recycling remains a big concern for the textile and fashion industry. Cotton is mainly composed of cellulose, which is a sustainable, renewable, and biocompatible natural polymer with versatile applications that currently utilised in many industries, including the paper and pulp industry and the food packaging industry [5]. Cellulose has also been employed in a variety of more advanced applications such as energy storage, drug delivery, and tissue engineering [6,7,8]. Since cotton itself is an excellent cellulose-rich feedstock, there is potential to exploit waste textile products and convert them into value-added cellulose-based products [9].

One such advanced product is cellulose beads, also known as cellulose particles or microspheres. It is a high-performance material with a wide range of applications [10,11,12]. Cellulose beads have a high porosity that make them suitable as absorbents for various substrates, such as heavy metal ions and enzymes for environmental remediation and the food industry, respectively [13,14]. Cellulose beads are also a promising filling material because of their spherical shape that leads to low flow resistance in chromatographic columns [11]. Compared with organic and inorganic based beads, cellulose beads have been shown to possess higher mechanical stiffness than silica-based beads and have a comparatively simple manufacturing process compared with synthetic polymer-based beads [10,15,16]. Additionally, cellulose beads are particularly promising for biomedical applications, including as carriers for drug molecules for oral delivery because of the biocompatibility of cellulose while achieving a high surface area, porosity, and low density of an aerogel [17]. In the literature, cellulose beads are usually categorised as a type of aerogel material with a spherical geometry because they have similar preparation procedures [16,18]. The preparation process for cellulose beads can be divided into three main steps: dissolution of cellulose, spheronisation, and coagulation [12]. Drying under special conditions is an optional step to preserve the porosity of beads in the dry state [19]. The size of cellulose beads can range from micrometres to a few millimetres, which is largely determined by the shaping method [20,21,22]. The spherical droplets are added into a bath that contains an anti-solvent for regeneration. The porous structure of cellulose beads is formed in this step through a continuous exchange between the dissolving solvent and anti-solvent [23]. The inner porous structure or morphology of cellulose beads is an important factor in determining its performance in various applications such as the loading capacity of drug molecules [24,25]. The most common morphologies that have been reported for cellulose aerogels are fibrous and globular structures. The fibrous morphology is composed of three-dimensional fibre-like entanglements that are inherited from the cellulose polymer chain, while the globular morphology is composed of globule-like aggregations [26,27]. The morphology can be tuned by altering the polymer concentration, the choice of dissolving solvent/anti-solvent, and the type of functionalisation applied to cellulose molecules [20,28,29].

In our previous study on cellulose aerogels [9], it was shown that the anion of the ionic liquid, which was used to dissolve the cellulose feedstock, had a prominent influence on the porous structure of cellulose aerogel. The major aim of the present study was to prepare cellulose beads from waste denim with different morphologies and porosity and to investigate the influence of the different morphologies on drug delivery efficiency.

## 2. Materials and Methods

### 2.1. Materials

Waste denim fabrics are made of 100% cotton. Sodium hydroxide (NaOH) (anhydrous pellets) was purchased from Sigma-Aldrich (Sydney, Australia). Two ionic liquids (ILs), 1-butyl-3-methylimidazolium chloride (BmimCl) and 1-butyl-3-methylimidazolium acetate (BmimAc) were purchased from IOLITEC, Heilbronn, Germany. Both ILs were used as received.

The two selected drugs, theophylline (Thp) and lidocaine hydrochloride monohydrate (LiHCl), were purchased from Sigma Aldrich. Distilled water was used as the solvent to prepare drug solutions.

### 2.2. Experimental Methodology

#### 2.2.1. Preparation of Cellulose Beads from Denim

**Figure 1:**
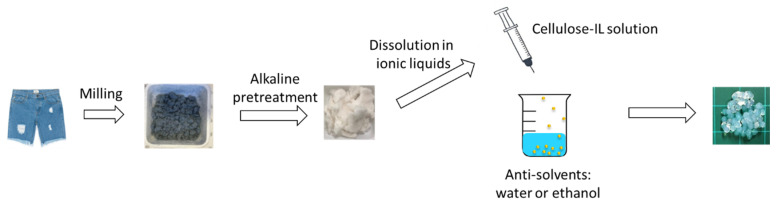
A schematic for the preparation of cellulose beads from waste denim.

As illustrated in Figure 1, denim fabrics were shredded and milled into 0.2-mm powders and pre-treated in aqueous NaOH solution (10% w/w) at 90 °C for 15 h. This pre-treatment reduced the molecular weight of denim and removed dirt. The obtained slurry was then rinsed with distilled water until NaOH was fully removed. The rinsed slurry was dried under 80 °C for 12 h [30].

The dissolution of denim in both ILs was conducted at 100 °C with magnetic stirring. The completeness of dissolution was verified by means of polarised microscopy when no crystalline structure could be observed. The denim concentration was fixed at 4 wt.% in this study.

The denim-IL solution was transferred into a 3-mL syringe and was extruded at a constant rate (manually). While the solution was being extruded from syringe nozzle, two methods were adopted for producing beads. For the cellulose-BmimAc solution, a blade was used to shear off the solution to form a bead. For cellulose-BmimCl, the solution was too viscous to be sheared off; therefore, scissors were used to cut the solution into spheres. The spherical shaped droplets were then dropped directly into a coagulation bath. Distilled water and ethanol were chosen as coagulation baths. The bath was changed every 1 h in the first 4 h and then immersed in water for 24 h. When ethanol was used as the coagulation bath, the obtained beads were washed with distilled water to exchange ethanol with water before drug loading. Table 1 lists the various cellulose beads prepared from waste denim and the solvent and coagulating solvent used.

#### 2.2.2. Characterisations of Cellulose–IL Solutions and Cellulose Beads

The rheological measurements of the cellulose–IL solutions were conducted on an ARES-G2 Rheometer (TA Instruments, New Castle, DE, USA) equipped with a Peltier temperature control system and a 40 mm parallel geometry. The gap was set at 50 mm for all samples. Oscillation frequency sweep measurements of the solution were conducted at 25 °C in the frequency range of 0.01 to 100 rad/s and the strain was set at 0.1%. Complex viscosity, storage modulus, and loss modulus were recorded.

The diameters of the fully coagulated beads were measured using a Vernier calliper and were controlled within 2.0 ± 0.1 µm. Thirty beads were measured for each type of beads. Supercritical CO_2_ drying was used to remove anti-solvents without influencing the solid content of cellulose beads so that the characterisations of the inner pore structure could be determined. All of the wet beads were washed with excessive ethanol to replace water and were then immersed in ethanol for 12 h before drying. The drying was performed on a Leica critical point drier (EM CPD300, Leica Microsystems, Wetzlar, Germany). The porosity of the supercritical-dried beads was analysed by N_2_ physisorption measurement on Quantachrome Autosorb iQ3 (Quantachrome Instruments, Boynton Beach, FL, USA). Samples were degassed at 80 °C for 10 h before being subjected to the 80-point physisorption measurements. The surface area was obtained by Brunauer–Emmett–Teller (BET) theory analysis on relative pressure 0.05–0.25, and the pore size distribution was analysed from the desorption curve based on the Barrett-Joyner-Halenda (BJH) theory with the exclusion of points with relative pressure below 0.3 [31,32]. Scanning electron microscopy (SEM) imaging was performed on Zeiss Supra 55V (Zeiss, Oberkochen, Germany) to observe the morphologies of cellulose beads. All the samples were coated with a 5-nm layer of gold before imaging.

#### 2.2.3. Drug Loading and In-Vitro Release

Lidocaine hydrochloride monohydrate (LiHCl) and theophylline (Thp) were selected as the representative substances, which are commonly employed for evaluating the drug delivery efficiency of different porous materials [25,33,34]. LiHCl is a type of amide-class local anaesthetics that is used to relieve nerve pain. Thp is a xanthine derivative, which is commonly used for asthma treatment. Thp molecules are immediately absorbed after oral administration, which relaxes the pulmonary blood vessels and the smooth muscles of bronchial airways and reduces the responsiveness of the airway to allergy-stimulating chemicals. Their solubilities in water, the concentrations used in the present study, and their indicative wavelengths in the UV-Vis spectrometer are listed in Table 2.

For each drug, a concentration measurement was carried out to correlate the absorption value with the solution concentration using the Cary 300 UV-Vis spectrometer (Agilent Technologies, Santa Clara, CA, USA). Four concentrations of drug solutions were prepared with 0.1 N HCl and the absorption values were recorded. The standard concentration curves of LiHCl and Thp are shown in Figures S1 and S2.

##### Drug Loading

The as-prepared wet beads were immersed in drug solutions for 24 h. The loaded beads were then removed from the drug solutions and dried in air overnight. The amount of incorporated drugs was obtained on the basis of the weights of dried beads before and after loading the drugs. Each batch involved 15 beads, and all the experiments were repeated three times. The loading capacity is calculated as follows:

(1) $$\mathrm{Loading}\;\mathrm{capacity}\;\left(\%\right)=\frac{weight\;of\;loaded\;beads-\mathrm{weight}\;\mathrm{of}\;unloaded\;beads}{weight\;of\;unloaded\;beads}\times 100.$$

##### In Vitro Drug Release

The dried loaded beads (15–25 beads in each batch) were immersed in 250-mL 0.1 N HCl buffer solution and constantly stirred at a fixed stirring rate of 100 rpm at 37.0 ± 0.5 °C. The release protocol is designed according to the USP paddle method with minor adjustments. The stirring paddle was maintained at the same position for each test. Four millilitres of the solution was taken for UV-Vis measurement at fixed five-minute time intervals for the first 20 min and then every 10 min from 20 to 80 min. The samples were taken at the same position each time to avoid deviations. All the experiments were performed in triplicate. The amount of the drug released was calculated using the obtained concentration–absorption curve. In the end, the drug release of each sample was plotted according to the percentage of the initial amounts versus time. The release efficiency is calculated as follows:

(2) $$\mathrm{Release}\;\mathrm{efficiency}\;\left(\%\right)=\frac{\mathrm{release}\;\mathrm{drug}\;\mathrm{amount}}{\mathrm{loaded}\;\mathrm{drug}\;\mathrm{amount}}\times 100$$

The swelling capacity of the beads was measured using a gravimetric method [35]. The dry beads were weighed and immersed in 0.1 N HCl solution for 2 h at 37.0 ± 0.5 °C. The swelled beads were gently wiped with Kimwipes and weighed.

## 3. Results and Discussion

**Figure 2:**
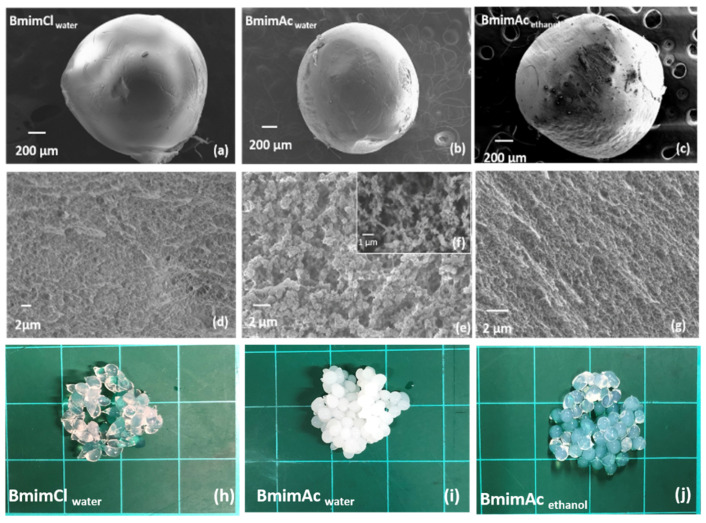
Scanning electron microscopy (SEM) images of surfaces (**a**–**c**) and cross sections (**d**–**g**) of cellulose beads prepared from denim; (**a**) and (**d**) Bmim**Cl**_water_; (**b**), (**e**) and (**f**) Bmim**Ac**_water_; (**c**) and (**g**) Bmim**Ac**_ethanol_. (**h**–**j**) present the appearances of cellulose beads.

Figure 2 shows the surfaces and cross sections of all three types of beads prepared from waste denim. It is observed that all the beads have a non-porous surface morphology. This is a commonly formed structure resulting from the rapid outflow of the solvent when the polymer solution is exposed to the anti-solvent [36]. The cross-sections of each bead showed the inner morphology differences; two distinctly different porous structures were obtained by varying the dissolving solvent. Bmim**Cl**_water_ has a cross-section that comprises a dense fibrous network at the nanoscale (Figure 2c). This fibrous structure is believed to be inherent from the intra- and inter-molecular hydrogen bonds of cellulose chains [16]. The cross-section of Bmim**Ac**_water_ (Figure 2e) appears to be composed of micro-sized globules, which are approximately 0.3~0.5 µm; these globular aggregates are fibrous at higher magnification (Figure 2f). This globular morphology has been reported in aerogels produced using various solvent systems and is regarded as the result of cellulose aggregations when hydrogen bonds are being rebuilt as the dissolving solvent leaves during coagulation [37]. These distinct morphological differences observed in the cellulose beads resulting from the use of different ILs as the dissolving solvents could be attributed to the difference in the solution state. Rheological measurements performed on both denim-IL solution samples at room temperature where coagulation occurs (Figure S3), show that the denim-Bmim**Cl** solution remains a liquid at room temperature but has much higher moduli than the denim-Bmim**Ac** solution. This indicates much lower mobility of cellulose molecules when dissolved in Bmim**Cl** compared with Bmim**Ac**, which may account for the fibrous morphology that forms under conditions when molecular aggregation is prevented due to slower coagulation kinetics. A simplified scheme is shown in Figure S4 for two systems. This explanation could also apply to previous observations of cellulose aerogels produced using aqueous solvent systems. In the literature, globular morphology has been observed when cellulose solutions are in the liquid state, while fibrous morphology appears when cellulose solutions are solidified or gelled when *N*-Methylmorpholine-N-oxide monohydrate and aqueous NaOH solution are used as the dissolving solvent [27,29].

The morphology can also be manipulated by using the anti-solvent (coagulating solvent); when ethanol was used as the coagulating solvent for the cellulose-BmimAc sample (Bmim**Ac**_ethanol_), a change from globular to fibrous network was observed, similar to that of Bmim**Cl**_water_ (Figure 2g). This morphological transition could indicate a slower diffusion rate of the IL molecules in ethanol compared with that in water because water was found to be a more effective anti-solvent than ethanol at breaking cellulose–IL bonds [38]. The reduced escaping speed of BmimAc may give rise to the steady rebuild of the hydrogen bonds among cellulose molecules, thus maintaining the intrinsic fibrous structure. The morphological differences also impact the beads’ appearance in terms of transparency, as shown in Figure 2. The two fibrous beads, Bmim**Cl**_water_ and Bmim**Ac**_ethanol_, are highly transparent, while Bmim**Ac**_water_ is opaque.

Table 3 presents the porosity and physical characterisations of each bead after supercritical drying, as measured by N_2_ physisorption. Bmim**Ac**_water_ has the lowest surface area among the three samples, and Bmim**Cl**_water_ shows a slightly higher surface area than Bmim**Ac**_ethanol_. The average pore size of the fibrous morphology (for both Bmim**Ac**_ethanol_ and Bmim**Cl**_water_) is the same (34.3 nm), which is significantly larger than the globular morphology (13.9 nm). This is likely due to the considerably higher diffusion rates of the solvent and anti-solvent in the case of Bmim**Ac**_water_. The cumulative pore volumes of the fibrous beads are consequently larger than that of globular beads with the larger average pore size. Between the two fibrous beads, Bmim**Ac**_ethanol_ has a higher cumulative pore volume than Bmim**Cl**_water_, thus indicating a higher porosity. As shown in Table 3, the diameters of all the beads are similar; however, differences in the weight of the beads in the dry state are noted. Beads prepared from Bmim**Ac**_water_ have a weight of 0.44 ± 0.04 mg, which is higher than that of Bmim**Ac**_ethanol_ (0.26 ± 0.01 mg) and Bmim**Cl**_water_ (0.35 ± 0.05 mg). This indicates a higher solid content in Bmim**Ac**_water_ than the other two beads and thus a lower porosity. This result is consistent with the different surface areas of the beads. Overall, the porosities of beads prepared from waste denim here are comparable with the reported cellulose beads and aerogels in various shapes, the pore sizes of which are mostly mesopores (2–50 nm), resulting in an surface area of 200–500 cm^3^/g [27,39].

**Figure 3:**
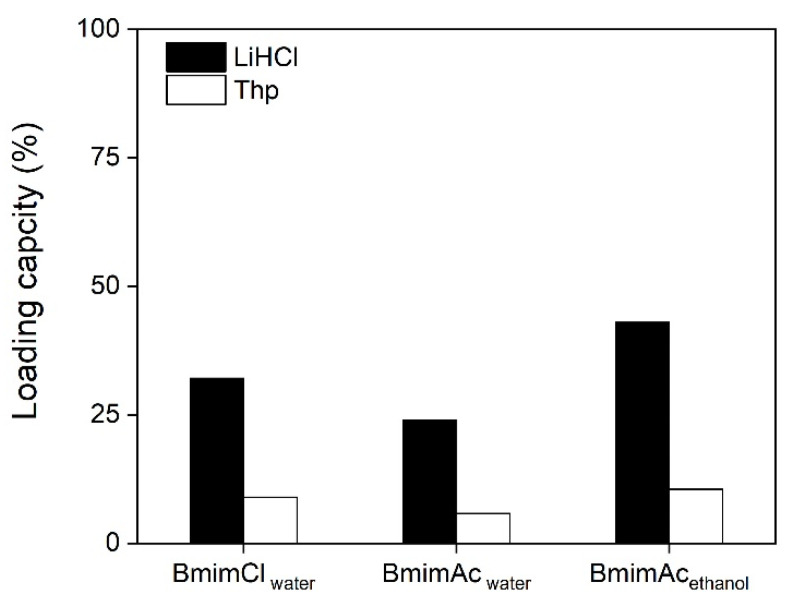
Loading capacities of cellulose beads prepared from waste denim.

The loading of the drug was achieved by immersing beads in the drug solution for 24 h. The amount of incorporated drug in the bead was determined by the weight difference of the dry bead before and after the drug loading. LiHCl and Thp were chosen as model drugs [25]. Because of their different solubilities in water, the concentration of each drug was different: 20 mg/mL for LiHCl and 4 mg/mL for Thp was used for the loading test. The loading capacity (%) was determined by the weight ratio of the amount of absorbed drug substrate to the weight of the unloaded dry bead, as shown in Figure 3. It can be seen that Bmim**Ac**_ethanol_ shows the highest loading capacity for both drugs, incorporating 43% and 10% for LiHCl and Thp, respectively (the loading capacity values for all beads loading are listed in Table S1). The significantly lower loading ratio of Thp when compared to LiHCl is due to the lower drug content used for Thp loading. Bmim**Cl**_water_ has a slightly lower loading capacity than that of Bmim**Ac**_ethanol_, which could be attributed to the lower surface area and smaller pore volume measured for Bmim**Cl**_water_. Bmim**Ac**_water_, with a globular morphology, shows much lower loading capacities than beads with fibrous morphology. Since Bmim**Ac**_water_ has the lowest pore volume, fewer loading sites for the drug molecules are present. This positive correlation between porosity (both surface area and pore volume) and loading capacity has been previously found in investigations of various drug delivery systems [25,40].

**Figure 4:**
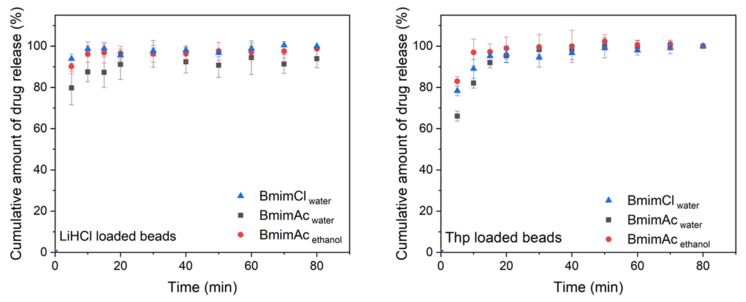
In vitro drug release curves for cellulose beads prepared from waste denim.

The loaded beads were then placed in 0.1 N HCl at 37.0 ± 0.5 °C, which mimics the stomach environment, to test their in vitro release profile over an 80-min period, as suggested by Yildir et al. [25]. The release curves are shown in Figure 4. It can be seen that regardless of morphology, the majority of the loaded drug was released in the initial five minutes followed by a steady release thereafter. This burst release is commonly observed and is due to the fact that most of the drug is absorbed on the surface of the bead [12]. Bmim**Ac**_water_ shows the lowest initial release for both drugs, which is likely due to a smaller average pore diameter. The smaller pore diameter could lead to more capillary condensation that enables more compact storage in the pores. When the beads are immersed in the suitable media, the absorbed drug molecules diffuse out in an ordered manner. The molecules that are stored inside the nanopores have to queue up, thus resulting in a sustained release behaviour. The release curves for Bmim**Ac**_ethanol_ and Bmim**Cl**_water_ are similar, as they both have a fibrous morphology. The swelling capacity of the beads is an important characteristic that influences the diffusion of the absorbed substrate. Table 4 lists the swelling capacities of three beads. It can be seen that in the beads with globular morphology, Bmim**Ac**_water_, has the lowest swelling capacity. This is consistent with the lower release profile, while both fibrous bead types have similar swelling capacities.

## 4. Conclusions

Three types of cellulose beads were prepared from waste denim with distinct morphologies and porosities by varying the IL used for dissolution and the coagulating solvent. By characterising the porous structure and examining the drug delivery profiles of three types of beads, it can be concluded that the loading capacities of the cellulose beads were positively correlated with the nanoporous characteristics of the beads (mainly the surface area and cumulative pore volume). Cellulose beads produced from BmimCl in water performed the best among the varieties, with 43% of their weight loaded with LiHCl. The subsequent drug release studies showed that 70%–90% of the loaded drug was released in the first five minutes across an 80-min measurement period. Surface functionalisation can be applied to cellulose to further improve the loading and release profiles of cellulose beads or achieve certain controlled release according to the desired performance for specific drugs and targeted releasing locations in the human body.

## Figures and Tables

**Table 1:** The ionic liquids (ILs) and coagulants used for the preparation of three types of cellulose beads.

Bead Name	Dissolving Solvent	Coagulant
Bmim**Cl**_water_	BmimCl	Water
Bmim**Ac**_water_	BmimAc	Water
Bmim**Ac**_ethanol_	BmimAc	Ethanol

**Table 2:** Two drugs selected for the drug delivery test.

Drug Name	Synonym	Solubility in Water (mg/mL)	Solution Concentration for Drug Loading (mg/mL)	Wavelength in UV-Vis (nm)
Lidocaine hydrochloride monohydrate (LiHCl)	2-(Diethylamino)-N-(2,6-dimethylphenyl)acetamide hydrochloride hydrate	147	20	218
Theophylline (Thp)	Dimethylxanthine	8	4	271

**Table 3:** Porosity characterisations of cellulose beads.

Bead Name	Specific Surface Area (m^2^/g)	Average Pore Size (nm)	Pore Volume (cm^3^/g)	Diameter of the Wet Beads (mm)	Diameter of Dry Beads (mg)	Weight of Dry Beads (mg)
Bmim**Cl**_water_	382	34.3	2.4	2.00 ± 0.08	0.75 ± 0.04	0.35 ± 0.05
Bmim**Ac**_water_	306	13.9	1.8	2.02 ± 0.08	0.80 ± 0.06	0.44 ± 0.04
Bmim**Ac**_ethanol_	348	34.3	3.7	2.00 ± 0.09	0.65 ± 0.04	0.26 ± 0.01

**Table 4:** Swelling capacities of cellulose beads prepared from waste denim.

	BmimCl_water_	BmimAc_water_	BmimAc_ethanol_
Swelling capacity	59.3%	48.3%	76.4%

## Supplementary Materials

The following are available online at https://www.mdpi.com/2073-4360/12/7/1621/s1; Figure S1: The standard concentration curve of LiHCl; Figure S2: standard concentration curve of Thp; Figure S3: rheological curves of denim-BmimCl and denim-BmimAc solutions at room temperature; Figure S4: Schematic explanation of cellulose mobility with two ILs; Figure S5: Pore size distributions of beads analysed from N_2_ physisorption. Table S1: loading capacities of LiHCl and Thp in the three types of beads.
