# Supplementary material for: Cellulose Beads Derived from Waste Textiles for Drug Delivery
Source: Polymers (Basel). 2020 Jul 21;12(7):1621. doi: 10.3390/polym12071621 (PMC7407133; doi:10.3390/polym12071621)
Supplement: Supplementary file 1 [file polymers-12-01621-s001.pdf]

Supporting information

Cellulose beads derived from waste textiles for drug delivery

Beini Zeng[a], Xungai Wang[a] and Nolene Byrne[a]

a Deakin University, Institute for Frontier Materials, Geelong Waurn Ponds Campus, Waurn Ponds, Victoria 3216, Australia.

Experimental methodology

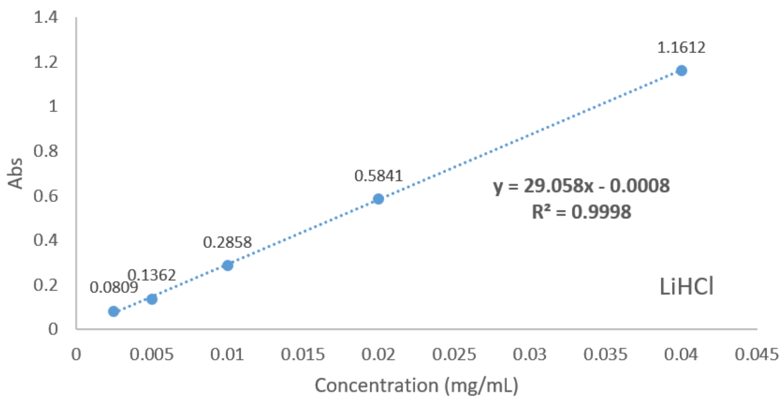

Figure S1. The standard concentration curve of LiHCl.

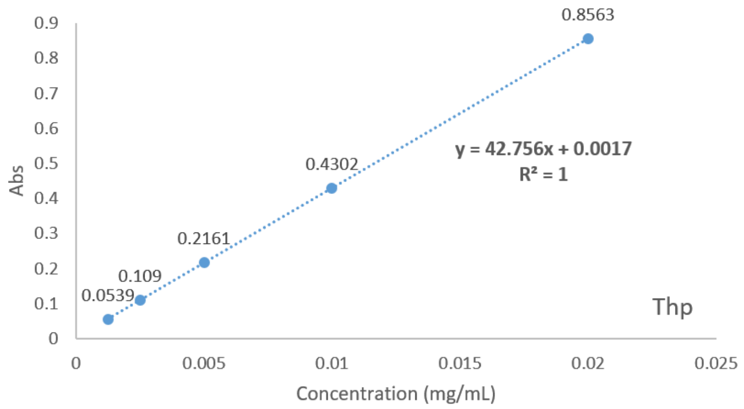

Figure S2. The standard concentration curve of Thp.

Results

Figure S3 shows the rheological characterisations of denim-BmimCl and denim-BmimAc solution. For both solutions, the storage modulus is higher than the loss modulus when the angular frequency is low, indicating a fluid-like state. However, the moduli of denim-BmimCl are much higher than that of denim-BmimAc, which indicates a higher flow resistance of denim-BmimCl.

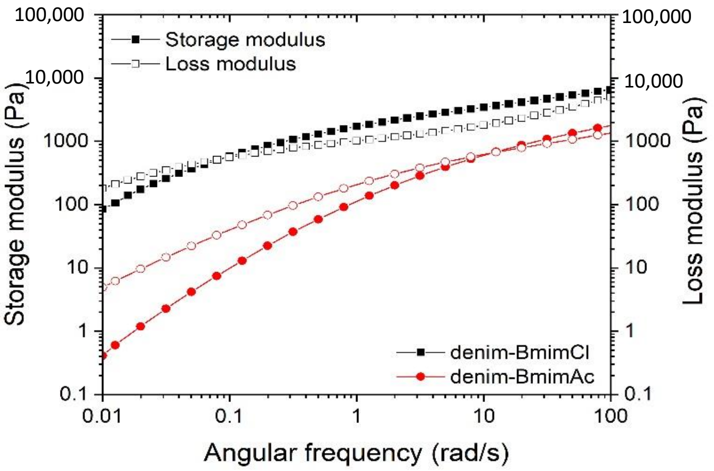

Figure S3. Rheological curves of denim-BmimCl and demo-BmimAc solutions at room temperature.

Figure S4 show a schematic explanation of the coagulation process of cellulose from two ionic liquids (ILs). It shows that the rigidity of BmimCl leads to a slight change in cellulose molecules before and after coagulation, whereas BmimAc provides more flexibility for cellulose molecules.

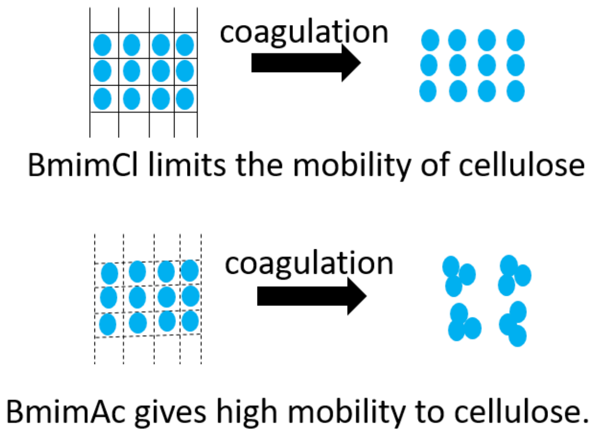

Figure S4. Schematic explanation of cellulose mobility in two ionic liquids (ILs) that leads to fibrous and globular morphologies. The blue sphere represents the cellulose molecules, and black lines represent the ILs.

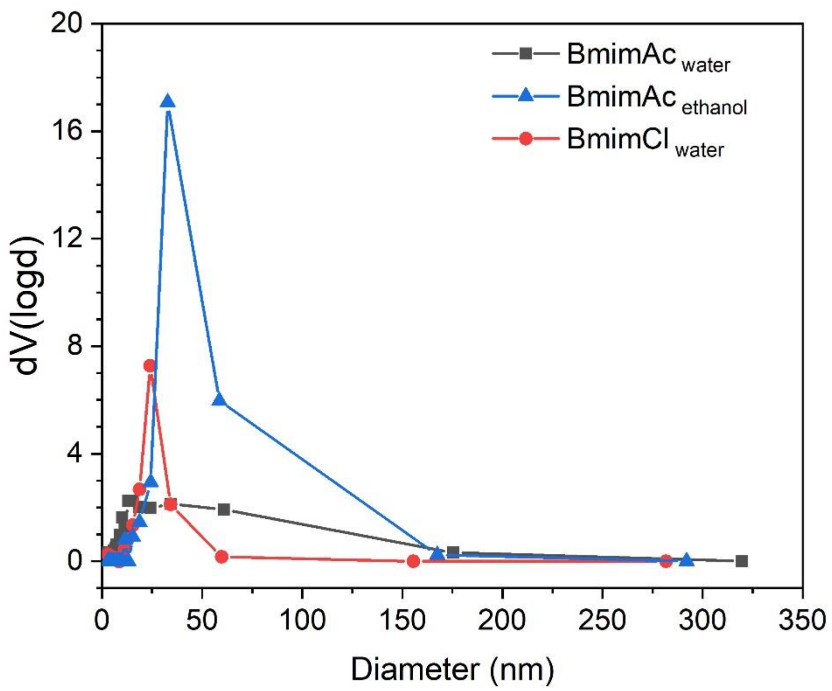

Figure S5. Pore size distributions of beads analysed from N_2 physisorption.

Table S1. Loading capacities of LiHCl and Thp in three types of beads.

**Bead type**	**Drug type**	**Loading capacity**
Bmim**Ac**$_{water}$	LiHCl	24.8%
	Thp	5.8%

Bmim**Ac**$_{ethanol}$	LiHCl	43.3%
	Thp	10.5%
Bmim**Cl**$_{water}$	LiHCl	32.1%
	Thp	9.0%
